# 2387. Assessment of COVID-19 primary series vaccination and factors associated with early and late uptake among Servicemembers in the Military Health System

**DOI:** 10.1093/ofid/ofad500.2007

**Published:** 2023-11-27

**Authors:** Laveta Stewart, Megan Clare Craig-Kuhn, Erica Sercy, Caryn Stern, Brock Graham, Amber Michel, Edward Parmelee, Stacy Shackelford, Simon Pollett, Timothy Burgess, David R Tribble, David R Tribble

**Affiliations:** Infectious Disease Clinical Research Program, Henry Jackson Foundation, Bethesda, Maryland; Infectious Disease Clinical Research Program, Department of Preventive Medicine and Biostatistics, Uniformed Services University of the Health Sciences; Henry M. Jackson Foundation for the Advancement of Military Medicine, Inc., Bethesda, Maryland; Infectious Disease Clinical Research Program, Department of Preventive Medicine and Biostatistics, Uniformed Services University of the Health Sciences; Henry M. Jackson Foundation for the Advancement of Military Medicine, Inc., Bethesda, Maryland; Joint Trauma System, JBSA Fort Sam Houston, Texas; Joint Trauma System, JBSA Fort Sam Houston, Texas; Infectious Disease Clinical Research Program, Department of Preventive Medicine and Biostatistics, Uniformed Services University of the Health Sciences; Henry M. Jackson Foundation for the Advancement of Military Medicine, Inc., Bethesda, Maryland; Infectious Disease Clinical Research Program, Department of Preventive Medicine and Biostatistics, Uniformed Services University of the Health Sciences, Bethesda, Maryland; Joint Trauma System, JBSA Fort Sam Houston, Texas; Infectious Disease Clinical Research Program, Department of Preventive Medicine and Biostatistics, Uniformed Services University of the Health Sciences, Bethesda, MD, USA, Bethesda, Maryland; Infectious Disease Clinical Research Program, Department of Preventive Medicine and Biostatistics, Uniformed Services University of the Health Sciences, Bethesda, MD, USA, Bethesda, Maryland; Uniformed Services University of the Health Sciences, Bethesda, Maryland; Uniformed Services University of the Health Sciences, Bethesda, Maryland

## Abstract

**Background:**

Identifying predictors of COVID-19 vaccination receipt in servicemembers (SM) is critical for protecting US military health. We report active-duty (AD) SM vaccination across 20 months of eligibility and assess factors associated with adoption of the primary series vaccine pre/post the Department of Defense (DoD) mandate, 8/24/21.

**Figure:**
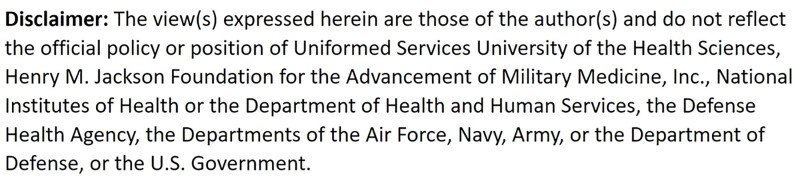

**Methods:**

Data on AD, National Guard/Reserve on AD, ages >17, Military Health System (MHS) beneficiaries (1/1/20 - 6/30/22) were extracted from the MHS Data Repository. Descriptive statistics were assessed using Chi-square. Univariate and multivariate modeling was conducted to examine the relationship of demographics and COVID-19 diagnosis with initiation of the COVID-19 primary series vaccine pre mandate. Statistical significance was defined as p< 0.05.

**Results:**

Of 1,889,750 SMs, most were ages 17-49, male, lived in the US, white, not Hispanic, and served in the Army. Overall, 90% received >1 primary series dose; with 85% completing the primary series, 5% had partial primary series, and 10% had no record of vaccination at the end of observed time.

Of SMs with >1 primary series dose, 78% received the 1st dose pre mandate (11% had COVID-19 prior to 1^st^ dose) and 22% post mandate (19% had COVID-19 prior to 1^st^ dose; p< 0.001) (Table 1 and Fig. 1).

SM ages >50 had higher odds of vaccine initiation pre mandate (P< 0.001), compared to ages 17-49 (Table 2). States in the Midwest and North central US had lower odds of pre mandate initiation compared to states in the southeast, aOR=0.85 and 0.87 respectively.

Black/African American SM had lower odds (aOR=0.71) of pre mandate initiation, and Asians or Pacific Islanders had higher odds (aOR=1.78) (P< 0.001) compared to white SM (Table 2). Coast Guard and Navy had higher odds of pre mandate initiation; Air Force and Marine Corps had lower odds (P< 0.001) compared to Army. SM who had COVID infection prior to 1^st^ primary dose had lower odds (P< 0.001) of initiating pre mandate.

**Table 1:**
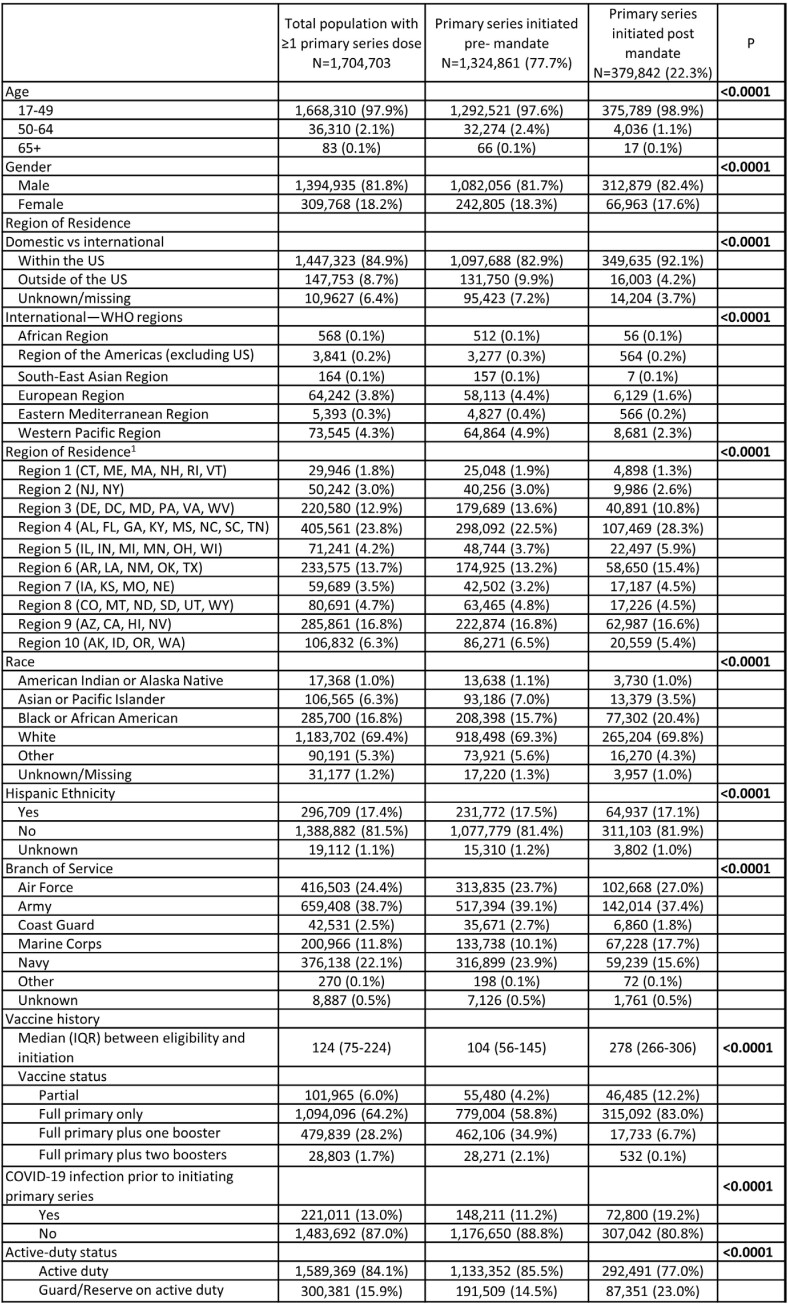
Demographic factors of the active-duty and Guard/Reserve on active-duty population who received ≥1 primary series vaccine dose according to primary series initiation timing (pre-mandate versus post-mandate)

1 Domestic regions of residence are defined by Health and Human Services (HHS) regions and all others are considered outside the US.

**Figure 1:**
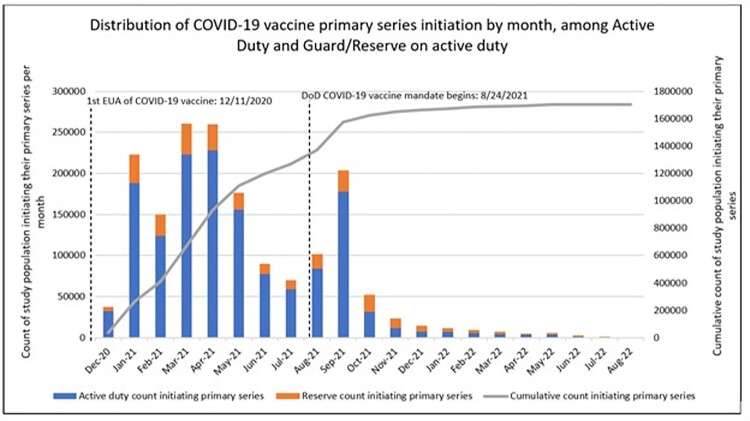
COVID-19 vaccine primary series initiation pre/post DoD mandate, by month among Servicemembers in the Military Health System

**Table 2.**
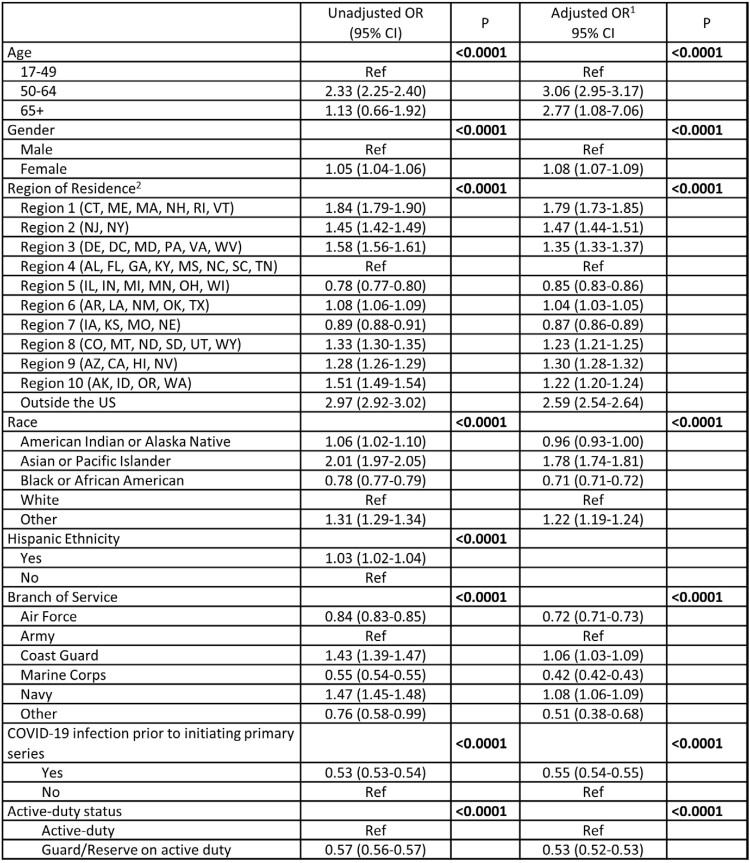
Univariate and adjusted associations with receiving a first primary dose prior to the COVID-19 vaccine military mandate (odds ratios>1 indicate higher odds of receiving the vaccine before the mandate)

1 The model was adjusted for all factors shown in the table: age, gender, region of residence, race, Hispanic ethnicity, branch of service, COVID-19 infection prior to initiation primary series, and active-duty status. 2 Domestic regions of residence are defined by Health and Human Services (HHS) regions and all others are considered outside the US.

**Conclusion:**

Although the majority of SM initiated the COVID-19 primary series prior to the DoD mandate, age, race, history of COVID-19 diagnosis, and region were associated with uptake post-mandate. Understanding the factors that affect vaccine uptake among SM is important to guide vaccine policy to enhance medical readiness and optimal vaccine effectiveness.

**Disclosures:**

**Simon Pollett, MBBS**, AstraZeneca: The IDCRP and the Henry M. Jackson Foundation (HJF) were funded to conduct an unrelated phase III COVID-19 monoclonal antibody immunoprophylaxis trial **Timothy Burgess, MD, MPH**, AstraZeneca: The IDCRP and the Henry M. Jackson Foundation (HJF) were funded to conduct an unrelated phase III COVID-19 monoclonal antibody immunoprophylaxis trial

